# Venlafaxine Improves the Cognitive Impairment and Depression-Like Behaviors in a Cuprizone Mouse Model by Alleviating Demyelination and Neuroinflammation in the Brain

**DOI:** 10.3389/fphar.2019.00332

**Published:** 2019-04-05

**Authors:** Yanbo Zhang, Xiaoying Bi, Olubunmi Adebiyi, Junhui Wang, Ali Mooshekhian, Jacob Cohen, Zelan Wei, Fei Wang, Xin-Min Li

**Affiliations:** ^1^ Department of Psychiatry, College of Medicine, University of Saskatchewan, Saskatoon, SK, Canada; ^2^ Department of Neurology, Changhai Hospital, Second Military Medical University, Shanghai, China; ^3^ Department of Physiology, Faculty of Medicine, University of Toronto, Toronto, ON, Canada; ^4^ Department of Pharmacology, College of Medicine, University of Saskatchewan, Saskatoon, SK, Canada; ^5^ Department of Psychiatry, The First Affiliated Hospital of China Medical University, Shenyang, China; ^6^ Department of Psychiatry, Faculty of Medicine and Dentistry, University of Alberta, Edmonton, AB, Canada

**Keywords:** venlafaxine, cuprizone, demyelination, cognition, major depressive disorder, oligodendrocyte, microglia

## Abstract

Growing evidence has implicated that myelin deficits and neuroinflammation are the coexisted pathological features that contribute to the mood swing and cognitive decline in major depressive disorder (MDD) and multiple sclerosis (MS). Therefore, attenuation of neuroinflammation and reduction of demyelination became newly emerging treatment strategies for the mood and cognitive symptoms. Antidepressant venlafaxine has been used in depression and anxiety through its multiple neuroprotective effects. However, it is unclear whether venlafaxine can improve myelin integrity and alter inflammation status in the brain. By using a well-established cuprizone-induced acute mouse model of demyelination, we investigated the protective effects of venlafaxine on these facets. The cuprizone-fed animals exhibited cognitive impairment and mood disturbances together with myelin loss and prominent neuroinflammation in the brain. Our present study showed that a high dose of venlafaxine alleviated the loss of myelin and oligodendrocytes (OLs), mitigated depression-like behaviors, and improved cognitive function in cuprizone-fed animals. Data from the present study also showed that venlafaxine reduced microglia-mediated inflammation in the brains of cuprizone-fed animals. These findings suggest that venlafaxine may exert its therapeutic effects *via* facilitating myelin integrity and controlling neuroinflammation, which may provide extra benefits to MS patients with depression and anxiety beyond the symptom management.

## Introduction

Major depressive disorder (MDD) affects around 300 million people worldwide and becomes a leading burden of the economy (Kassebaum et al., 2016). The core symptoms of MDD include depressed mood, lack of interest, difficulties in concentration, changes in appetite and sleep, and cognitive impairment (APA, 2013). The treatment for depression is challenging as approximately 30% of MDD patients develop treatment-resistant depression (TRD) despite subsequent antidepressant augmentations or switches (Rush et al., 2006). The development of TRD is mainly due to our limited understanding of the neurobiology of MDD and the action mechanism of antidepressant treatment (Huezo-Diaz et al., 2005). It was supposed that antidepressants exert their acute effects primarily by increasing the availability of serotonin (5-HT), norepinephrine (NE), or both in the synaptic cleft (Berton and Nestler, 2006). Although antidepressants immediately increase the monoamine levels in the brain, it takes at least 2–3 weeks for the occurrence of mood-enhancing effects, thereby indicating other mechanisms may contribute to its efficiency.

MDD is considered a complex brain disorder resulting from abnormal brain structural and functional connectivity (Gong and He, 2015). White matter tracts are critical for interconnecting brain regions and transferring the neural activities essential for organizing human behavior, emotions, and cognition (Filley and Fields, 2016). Neuroimaging studies have shown white matter abnormalities in the brains of patients with MDD (Yamada et al., 2015; Cyprien et al., 2016). Oligodendrocytes (OLs) are residential glial cells responsible for generating myelin sheaths and white matter tracts in the central nervous system (CNS) (Nave and Werner, 2014). Abnormalities in myelin and OL are associated with cognitive impairment and increased suicide attempts in MDD patients (Yamada et al., 2015; Cyprien et al., 2016).

Multiple sclerosis (MS) is a demyelinating inflammatory disorder. Over half of MS patients exhibit MDD comorbidity (Feinstein, 2004), thereby suggesting some biological abnormalities (e.g., deficits of myelin and OL and neuroinflammation) may coexist in MDD and MS (Morris et al., 2018). While a variety of treatments including antidepressants, psychotherapy, and neuromodulation have shown therapeutic effects due to the improvement of the white matter structure and function (Mostert et al., 2006; Zeng et al., 2012; Wang et al., 2013; Anderson et al., 2016), cases with severe white matter changes showed poor responses to antidepressant treatment (Peng et al., 2013; Serafini et al., 2015). Together, these findings suggest white matter/myelin integrity is a new treatment target for MS and MDD, especially for treatment-resistant depression (Serafini et al., 2015).

Venlafaxine, a serotonin and noradrenaline reuptake inhibitor (SNRI) antidepressant, has been widely used for MDD, anxiety, and neuropathy (Tundo et al., 2015; Waldfogel et al., 2017). Venlafaxine is also a preferred choice for monotherapy or combination treatment for TRD (Tundo et al., 2015). Studies found that venlafaxine possesses the neuroprotective effects *via* its anti-inflammatory activities (Xu et al., 2006; Chen et al., 2018). Our previous research showed that desvenlafaxine, a major active metabolite of venlafaxine, prevented stress-induced white matter injuries in mice (Wang et al., 2014). However, it is unknown whether venlafaxine can provide neural protections by acting on OLs and preserve myelin integrity. The cuprizone-induced demyelinated mice showing white matter deficits and cognitive and emotional impairments with minimal motor function deficits (Zhang et al., 2012; Yan et al., 2015) are a suitable animal model in our present study to explore the effects of venlafaxine on behavioral abnormalities, myelination/OL deficits, and neuroinflammation.

## Materials and Methods

### Animals

Seven-week-old female C57BL/6 mice were purchased from Charles River (Montreal, Canada) and hosted in the animal facility maintained at a 12-h/12-h light–dark cycle, at 22 ± 0.5°C and 60% humidity, with *ad lib* to food and water. All animal procedures were performed under the Canadian Council on Animal Care (CCAC) guidelines and were approved by the University Committee on Animal Care and Supply (UCACS) of the University of Saskatchewan. Mice develop selective central demyelination and inflammation in the prefrontal cortex, hippocampus, and the corpus callosum (CC) after 5 weeks of CPZ treatment (Zhang et al., 2008).

### Drug Treatment

Cuprizone (CPZ, Sigma-Aldrich, St. Louis, MO, USA) was mixed into the milled Lab Diet rodent chow (PMI Nutrition International LLC, Brentwood, MO, USA) with a final concentration of 0.2% (w/w), as previously described (Zhang et al., 2008). Venlafaxine (Pfizer, Montreal, Canada) was dissolved in distilled water. After acclimatization with the regular rodent diet for 1 week, mice were divided into six groups (16 mice per group). Mice in the first three groups received regular chows plus daily treatment with either water (CTL), or 5 mg/kg/day of venlafaxine (VEN5), or 20 mg/kg/day of venlafaxine (VEN20) for 5 weeks. The remaining groups received rodent chows containing 0.2% CPZ (w/w) plus daily treatment with either water (CPZ), or venlafaxine 5 mg/kg/day (CPZ + VEN5), or venlafaxine 20 mg/kg/day (CPZ + VEN20) for 5 weeks. Body weight was measured twice weekly. Behavioral tests were performed to evaluate depression-like behaviors and working memory during the fifth week.

#### Behavioral Tests

The locomotor activity, spatial working memory (Y-maze spontaneous alternation), and depression-like behaviors such as tail suspension test (TST) and forced swim test (FST) were performed during the fifth week after treatment. Only one behavioral test was carried out in each day with the order like this: (1) locomotor activity, (2) Y-Maze, (3) the first FST, (4) TST, and (5) the second FST.

##### Locomotor Activity Test

The spontaneous locomotor activity was measured using a light beam system as described previously (Zhang et al., 2012). Briefly, each mouse was put in a transparent cage (40 cm × 40 cm × 25 cm) equipped with photo beams near the bottom of the cage. After 1-min adaptation, the frequency of photo-beam interruptions during the subsequent 5-min period was recorded to measure total movements, including both horizontal and vertical movements.

##### Y-Maze Test

The working memory was assessed by recording spontaneous alternation in a Y-maze apparatus. It is the natural tendency of rodents to explore a novel environment (Lamberty et al., 1992). The normal mice will remember the arm they have already explored and will enter one of the other arms of the maze. The Y-maze has three arms, named A, B, and C. Mice were placed individually onto the end of one arm (A) and allowed to explore all three arms freely for 8 min. After the first 2 min for habituation, the alternations between arms were recorded during the remained 6 min using Anymaze^™^ behavioral analyzing software (Hughes, 2004). The alternation defined as the overlapping entrance sequence (e.g., ABC, BCA) was calculated as the percentage: alternation % = (number of alternations)/(total number of arm entries − 2) × 100 (Zhang et al., 2008). Total arm entries were used as an index of ambulatory activity. Mouse with five or fewer arm entries in 6 min was excluded from the data analysis.

##### TST and FST

FST and TST have been commonly employed to evaluate the depression-like behaviors and antidepressant efficacy in the rodent models (Porsolt et al., 1977). In these tests, a mouse was submitted to an inescapable aversive situation, alternating periods of activity and immobility to reflect “behavioral despair” (Steru et al., 1985). In FST, mice were placed in a Plexiglas cylinder (10 cm internal diameter, 20 cm in height) filled with water (25–26°C temperature and 10 cm in height). Each mouse was allowed to swim for a 6-min session. The total immobility period during the last 4 min of the session was recorded. The immobility period was defined as the time while mice were utterly inactive or made movements only necessary to keep their heads above water. In TST, mice were suspended for 6 min in the air by taping their tails on a hanging bar. The immobility time during the 6 min was recorded. The immobility period was defined as the time while mice displayed no movement when suspended on the bar. All behaviors were recorded using a digital camcorder. The videos were analyzed by an observer blind to treatments using Anymaze^™^ behavioral software.

### Histological and Immunohistochemical Staining

After behavioral tests, all mice were euthanized with sodium pentobarbital (50 mg/kg, i.p.) and perfused intracardially with phosphate-buffered saline (PBS) followed by 4% paraformaldehyde (PFA) in PBS. Brains were then fixed overnight in 4% PFA. The fixed brains were subsequently rinsed three with PBS and cryoprotected in 30% sucrose in PBS at 4°C for 36 h. Three coronal sections (30 and 300 μm apart) between levels 1 and −1 mm from bregma were used for histological or immunohistochemistry staining. Luxol fast blue periodic acid-Schiff (LFB-PAS) (Sigma, St. Louis, MO, USA) staining was used to detect the severity of white matter demyelination in the CC (Pappas, 1981; Lin et al., 2005). For immunohistochemical staining, floating brain sections were quenched for half hour in PBS with 0.3% hydroperoxide, followed by 1-h incubation in blocking solution containing 10% host serum in PBS at room temperature (RT). The sections were subsequently incubated overnight with the primary antibodies diluted in the blocking solution and incubated with the anti-goat or anti-rabbit biotin-conjugated secondary antibody (1:1,000; Vector Laboratories, Burlingame, CA) for 2 h at RT. The avidin-biotin complex kit (Vector Laboratories, Burlingame, CA) and 3,3-diaminobenzidine (DAB) chromogen (Sigma-Aldrich, St. Louis, MO) were used to visualize the staining. The brain sections were incubated at RT with the Alexa Fluor fluorescence secondary antibodies for immunofluorescent staining.

### Antibodies

Goat polyclonal antibody directed against myelin basic protein (MBP) (1:250; Santa Cruz Biotechnology, CA) was used to detect the myelin protein component (Zhang et al., 2008). Rabbit anti-Olig2 (1:200) and anti-nerve/glial antigen 2 (NG2) antibodies (1:200) (Millipore, Temecula, CA) were used as markers for cells in OL lineage and oligodendrocyte progenitor cells (OPCs), respectively (Zhang et al., 2012). Rat anti-glutathione S-transferase isoform π (GST-π, 1:500; Stressgen, Victoria, BC, Canada) was used to identify the mature OLs (Zhang et al., 2012). Olig2 and GST-π double labeling enabled to identify a subgroup of OL lineage cells with negative GST-π staining; these cells are considered either OPCs or immature OLs (Zhang et al., 2008). Rabbit polyclonal anti-CD11b (1:500; AbD Serotec, Raleigh, NC, USA) and goat anti-glial fibrillary acidic protein (GFAP) antibodies (1:1,000; Sigma, St. Louis, MO, USA) were used to identify activated microglia and astrocytes, respectively (Zhang et al., 2008).

### Image Analysis

All images were obtained using an Olympus BX-51 light microscope or Olympus Confocal Laser Microscope 510 Meta. For each immunostaining analysis, digital images from three coronal sections were analyzed by two researchers blinded to the treatment using ImageJ software (version 6.1, Media Cybernetics, Inc., Silver Spring, MD). The software settings for imaging were kept identical among brain sections in each immunostaining. Demyelination in the CC was determined using a modified semiquantitative scale system with 0–4 points (Das Sarma et al., 2009): 0 (no demyelination), 1 (rare and focal demyelination), 2 (multiple focal demyelination), 3 (large or confluent demyelination), and 4 (large and confluent demyelination over 75% of CC). Demyelination in the cortical area was evaluated and expressed as the percentage of MBP-positive staining in a selected area vs. the staining in the corresponding area from control groups. GST-π and Olig2 immunostainings were measured from randomly selected areas within the CC in each section to quantify mature OLs and OL lineage cells, respectively. The cell numbers of CD11b-, GFAP-, and NG2-positive cells in the CC were evaluated using ImageJ software. Results were presented as the mean number of positive cells per square millimeter. Results from each animal were counted in three coronal sections, and the data are presented as the average of 16 mice per group.

### Statistical Analysis

Group differences were determined using two-way analyses of variance (ANOVA). Bonferroni *post hoc* analyses assessed statistical significance between groups. The nonparametric data were analyzed by the Kruskal-Wallis test and follow-up Dunn’s multiple comparison test. The results were expressed as mean ± SEM. *p* < 0.05 was considered statistically significant.

## Results

### Effects of Venlafaxine on CPZ-Induced Behavioral Deficits

#### Locomotor Activity

**Figure 1:**
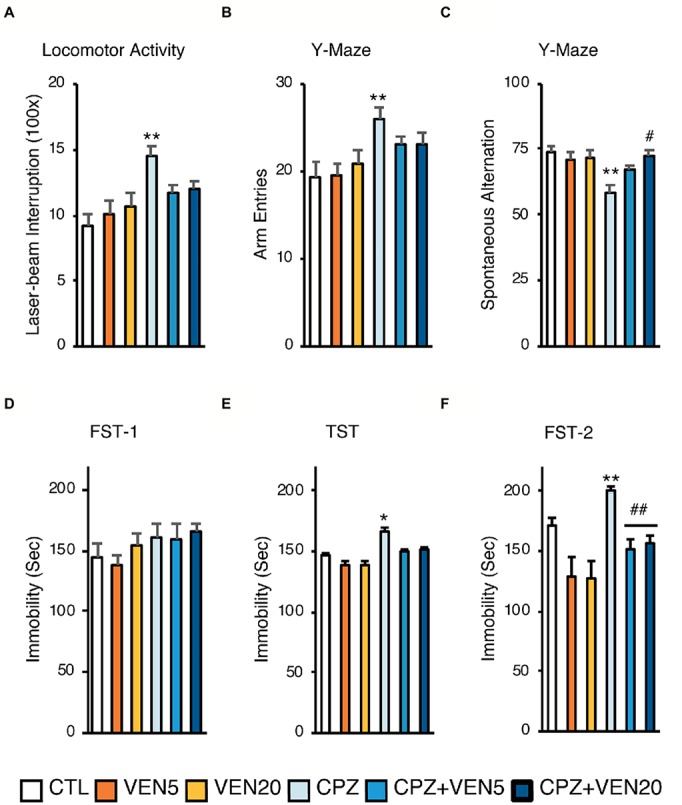
The effects of venlafaxine on behaviors in normal and demyelinated mice. All mice were tested for **(A)** locomotor scores in the open field; **(B)** arm entries in the Y-maze test; **(C)** spontaneous alternative in the Y-maze test; **(D)** immobile time during the first trial of forced swim tests (FST-1); **(E)** immobile time during tail suspension test (TST); **(F)** immobile time during the second trial of forced swim tests (FST-2). Data were presented as means ± SEM (*n* = 16 in each group). *p < 0.05 or **p < 0.01 compared to the CTL; ^#^*p* < 0.05 or ^##^*p* < 0.01 compared to CPZ. CTL, control; CPZ, cuprizone; VEN5, venlafaxine 5 mg/kg/day; VEN20, venlafaxine 20 mg/kg/day.

As previously reported, mice receiving 5-week CPZ diet exhibited locomotor hyperactivity in a novel environment (Xu et al., 2009; Bustillo et al., 2010). We further examined whether venlafaxine treatment would moderate the behavioral abnormality. Locomotor activity level was measured by the frequency of photo-beam interruptions in a 5-min test period. Two-way ANOVA showed a main effect of CPZ (*F* = 15.76, *p* = 0.0002); CPZ exposure significantly increased the frequency of photo-beam interruption compared to regular diet (Figure 1A). Mice exposed to CPZ also had higher total arm entries in the Y-maze test compared to those with the regular diet (*F* = 15.57, *p* = 0.00021). Importantly, two-way ANOVA yielded a model-by-treatment interaction (*F* = 4.04, *p* = 0.02). Venlafaxine showed no effects on the frequencies of photo-beam interruption or arm entries (*p* > 0.05) (Figures 1A,B).

#### Working Memory

There was a significant model-by-treatment interaction (*F* = 4.54, *p* = 0.015) in the Y-maze spontaneous alternation test. CPZ mice displayed a substantial spatial memory deficit compared to the CTL group (*p* < 0.003) (Xu et al., 2009). The *post hoc* Scheffe’s test showed a significantly lower alternation in the CPZ group (54.0%) compared to the CTL group (80.7%). Notably, there was a dose-dependent effect of venlafaxine on CPZ-induced spatial memory deficit, showing that only a higher dose was effective (CPZ + VEN20, *p* = 0.02) (Figure 1C).

#### Depression-Like Behaviors

In the first FST (FST-1), there was no significant difference in immobility time among groups (Figure 1D), suggesting that neither venlafaxine nor CPZ elicited “despair behaviors” after 5-week treatment. Two-way ANOVA revealed significant model-by-treatment interaction in the TST on day 2 (*F* = 3.41, *p* = 0.03). The CPZ group had a significantly prolonged immobility time compared to the CTL group (*p* = 0.0137). There were no differences among CTL and venlafaxine suggesting that venlafaxine reversed the prolonged immobility time caused by CPZ (Figure 1E). Interestingly, there was a significant model-by-treatment interaction (*F* = 4.24, *p* = 0.02) and a main effect for CPZ (*F* = 8.84, *p* = 0.005) in the second FST (FST-2). The CPZ groups had a significantly longer immobility time compared to CTL group (*p* = 0.00063); alternatively, venlafaxine treatments decreased the immobility times compared to CPZ group (CPZ + VEN5 vs. CPZ, *p* = 0.015, CPZ + VEN20 vs. CPZ, *p* = 0.035) (Figure 1F).

### Effects of Venlafaxine on CPZ-Induced Demyelination in the CC and the Cerebral Cortex

**Figure 2:**
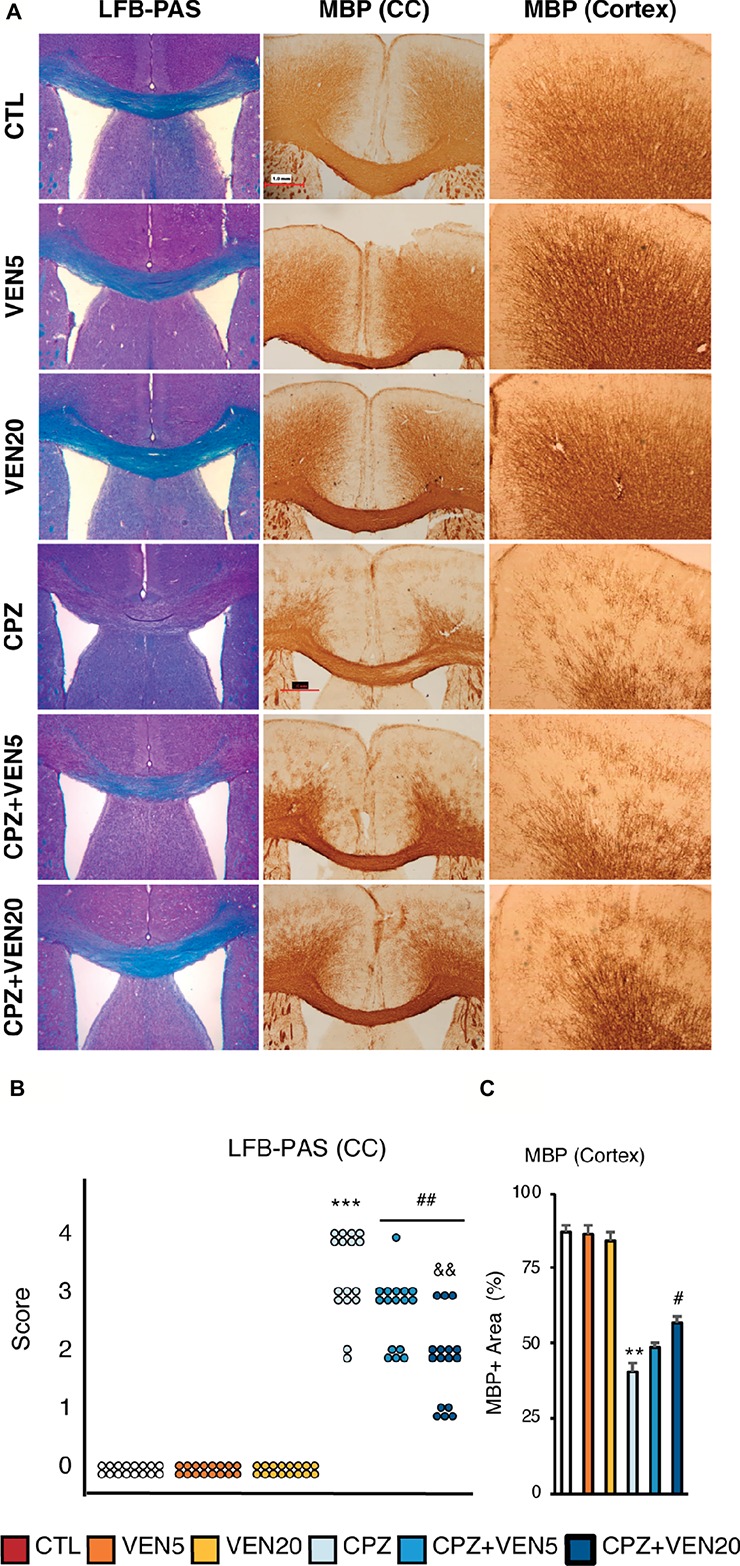
The effects of venlafaxine on myelin integrity in normal and demyelinated mice. **(A)** Representative images of Luxol fast blue and periodic acid-Schiff (LFB-PAS) staining in the corpus callosum (CC) and myelin basic protein (MBP) immunostaining in the CC and cortex; **(B)** demyelination score based on LFB-PAS staining in the CC; **(C)** Quantitative analysis of cortical demyelination measured by percentage of MBP immunostaining area in the frontal cortex in each group. Data are presented as mean ± SEM (*n* = 16 mice per group). The LFB-PAS score was analyzed by the Kruskal-Wallis test (Dunn’s multiple comparison test). MBP staining data were analyzed by two-way ANOVA followed by Bonferroni *post hoc* analyses. Significances among different conditions are indicated as below: **p < 0.01 or ***p < 0.001 compared to the CTL; ^#^*p* < 0.05 or ^##^*p* < 0.01 compared to CPZ. ^&&^*p* < 0.01 compared to CPZ + VEN5. Scale bar represents 100 μm; CTL, control; CPZ, cuprizone; VEN5, venlafaxine 5 mg/kg/day; VEN20, venlafaxine 20 mg/kg/day.

Five-week CPZ treatment causes extensive demyelination in the CC and the cerebral cortex of C57BL/6 mice (Xu et al., 2009) (Figure 2A). The LFB-PAS staining showed significantly higher demyelination scores in the CC in CPZ compared to CTL groups in the CC (Figure 2B, *p* < 0.0001). Venlafaxine in the regular diet mice had no effect on the myelination status. On the contrary, a high dose of venlafaxine (20 mg/kg/day) significantly alleviated CPZ-induced demyelination in the CC, while lower dose of venlafaxine (5 mg/kg/day) failed to show any improvement (Figure 2B). The percentage of MBP-positive stained area showed that mice exposed to CPZ had a significant loss of MBP compared to the CTL mice in the cerebral cortex; venlafaxine treatment did not change the percentage of MBP staining in mice on the regular diet. High dose of venlafaxine (20 mg/kg/day) significantly reduced the cortical demyelination caused by CPZ (*p* < 0.05). Altogether, these findings suggest that venlafaxine exerted neuroprotective effects on myelin sheaths in both the CC and cortical areas against CPZ-induced demyelination in a dose-dependent fashion (Figure 2C).

### Effects of Venlafaxine on Oligodendrocytes in the CPZ-Induced Demyelination

**Figure 3:**
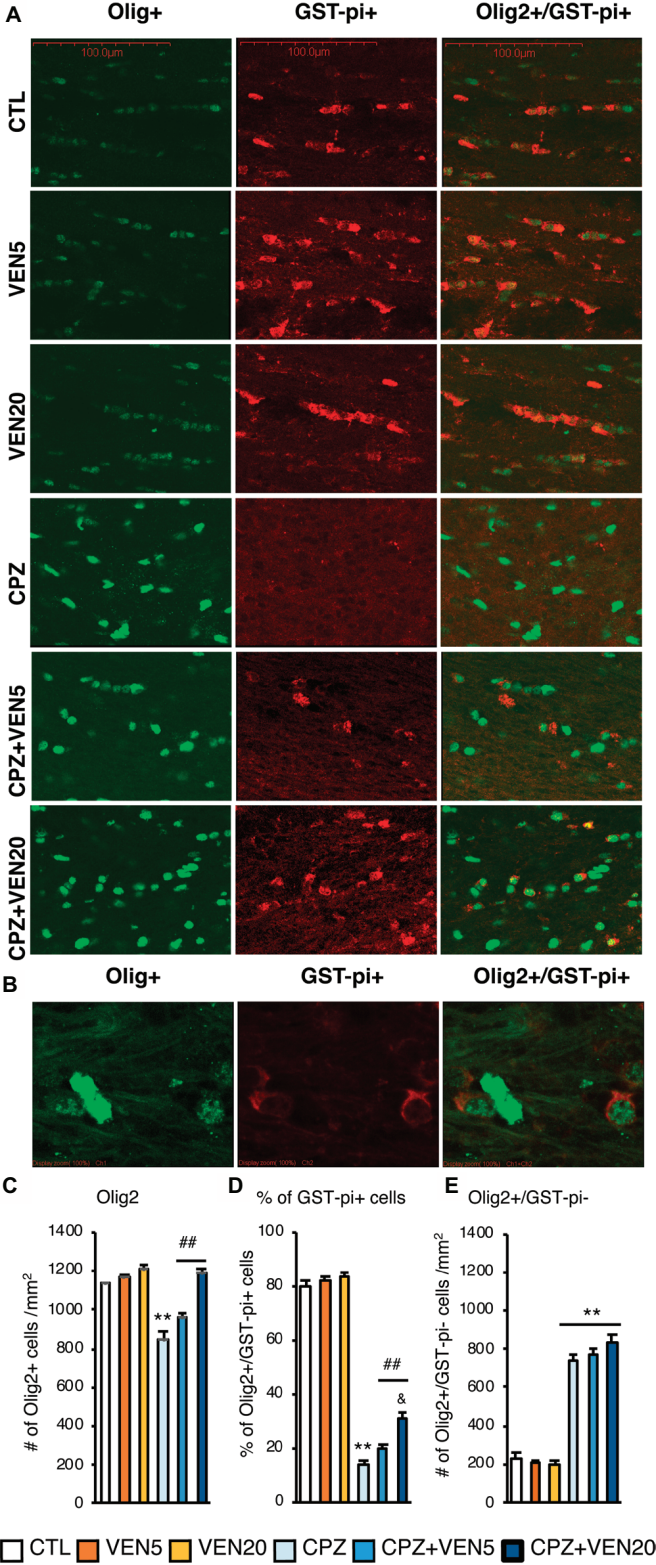
Venlafaxine reduced mature OL loss in the corpus callosum (CC) of cuprizone-exposed C57BL/6 mice. **(A)** Representative images of Olig2, GST-π, and Olig2/GST-π double labeling in the CC areas; **(B)** the double labeling of Olig2 and GST-π at high magnification; **(C)** quantitative analysis of the total number of Olig2+ cells; **(D)** the percentage of Olig2+/GST-π+ cells in the CC in each group; **(E)** quantitative analysis of the Olig2+/GST-π− cells in the CC in each group. Data are presented as mean ± SEM (*n* = 16 mice per group). The data were analyzed by two-way ANOVA followed by Bonferroni *post hoc* analyses. Significances among different conditions are indicated as below: **p < 0.01 compared to the CTL; ^##^*p* < 0.01 compared to CPZ and ^&^*p* < 0.05 compared to CPZ + VEN5. Scale bar represents 100 μm; CTL, control; CPZ, cuprizone; VEN5, venlafaxine 5 mg/kg/day; VEN20, venlafaxine 20 mg/kg/day.

**Figure 4:**
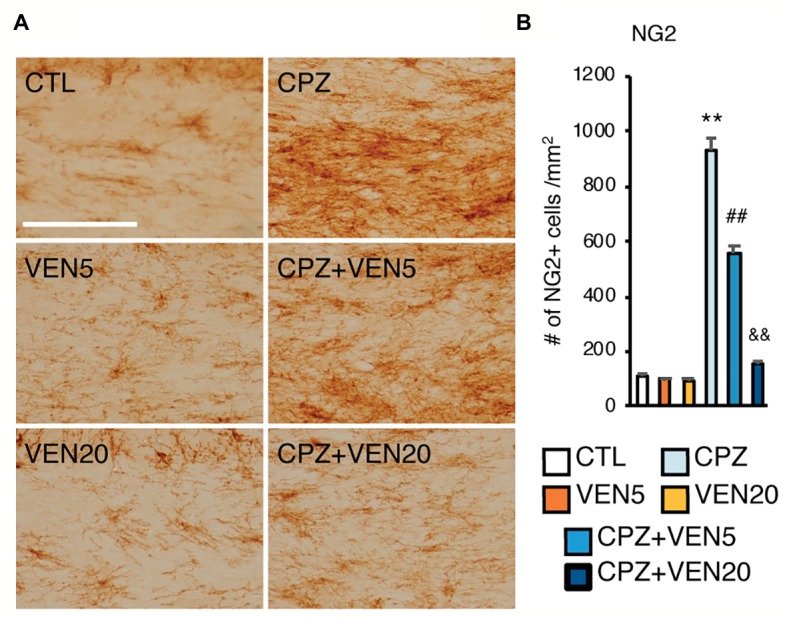
Venlafaxine decreased oligodendrocyte (OL) progenitor cell (OPC) accumulation in the corpus callosum (CC) of cuprizone-exposed C57BL/6 mice. **(A)** Representative images of NG2 immunostaining in the CC areas; **(B)** quantitative analysis of the number of NG2+ cells in the CC in each group. The results are presented as mean ± SEM (*n* = 16 mice per group). The data were analyzed by two-way ANOVA followed by Bonferroni *post hoc* analyses. Significances among different conditions are indicated as below: **p < 0.01 compared to the CTL; ^##^*p* < 0.01 compared to CPZ, and ^&&^*p* < 0.01 compared to CPZ + VEN5. Scale bar represents 100 μm; CTL, control; CPZ, cuprizone; VEN5, venlafaxine 5 mg/kg/day; VEN20, venlafaxine 20 mg/kg/day.

The number of total OL lineage cells (Olig2+) in the CC was significantly reduced in the CPZ and CPZ + VEN5 groups, compared to the CTL groups (Figures 3A–C); however, CPZ + VEN20 groups demonstrated a significantly higher number of Olig2+ cells compared to the CPZ and CPZ + VEN5 groups (Figures 3A–C). The Olig2+ cells were further categorized into GST-π− and GST-π+ cells using Olgi2 and GST-π double labeling. The majority of Olig2+ cells (~80%) were mature OLs (GST-π+) in the CC in CTL groups; there was only 15% of Olig2+ cells were GST-π+ in CPZ group. A high dose of venlafaxine significantly increased the percentage of the GST-π+ cells to 35% (Figure 3D). The Olig2+/GST-π− cells were significantly increased in all CPZ groups, and venlafaxine seemed did not alter the cell numbers in CPZ groups (Figure 3E). The number of NG2+ OPCs was significantly increased in CPZ-exposed mice compared to CTL groups (*p* < 0.001). Conversely, venlafaxine treatment significantly reduced the number of NG2+ in the CC in the CPZ-exposed mice (Figure 4A). A high dose of venlafaxine was more efficient compared to a low dose of venlafaxine (Figure 4B).

### Venlafaxine Reduces Inflammatory Changes in the CC

**Figure 5:**
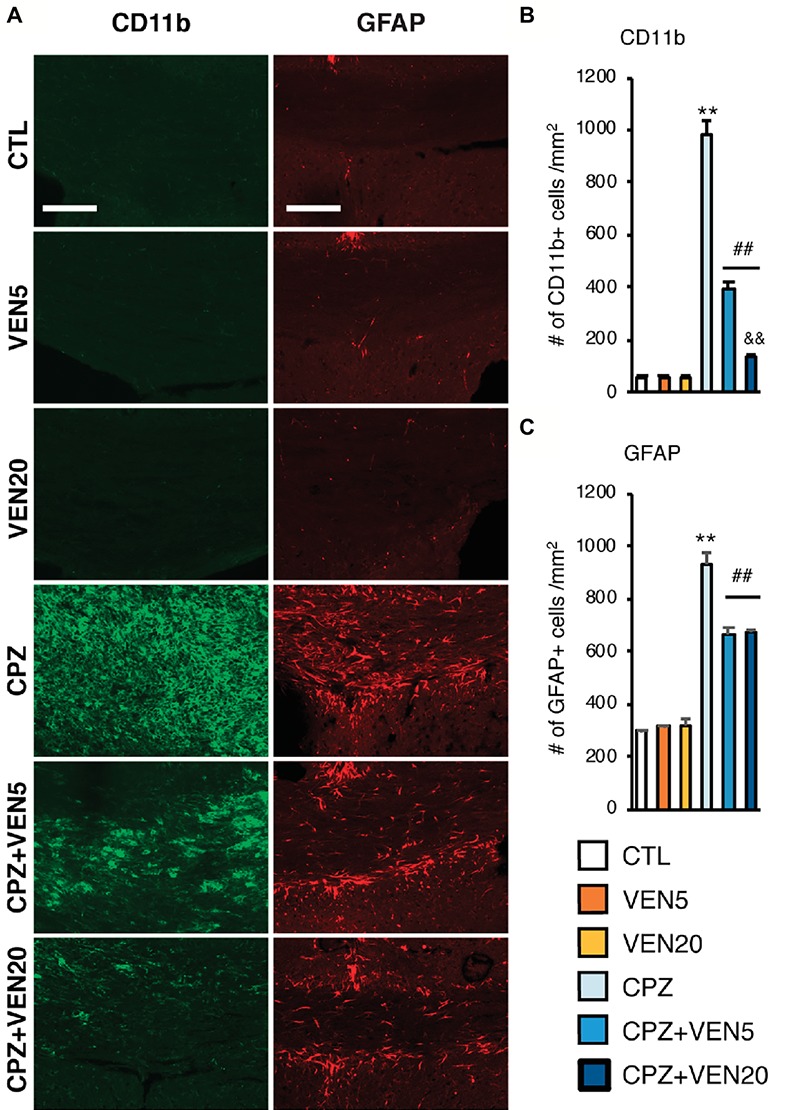
Venlafaxine inhibited microglia and astrocyte activation in the corpus callosum (CC) of cuprizone-exposed C57BL/6 mice. **(A)** Representative images of CD11b and GFAP immunostaining in the CC areas; **(B)** quantitative analysis of the cell number of CD11b+ cells in the CC in each group. **(C)** Quantitative analysis of the cell number of GFAP+ cells in the CC in each group. Data are expressed as mean ± SEM (*n* = 16 mice per group). The data were analyzed by two-way ANOVA followed by Bonferroni *post hoc* analyses. Significances among different conditions are indicated as below: **p < 0.01 compared to the CTL; ^##^*p* < 0.01 compared to CPZ, and ^&&^*p* < 0.01 compared to CPZ + VEN5. Scale bar represents 100 μm; CTL, control; CPZ, cuprizone; VEN5, venlafaxine 5 mg/kg/day; VEN20, venlafaxine 20 mg/kg/day.

In the present study, we used CD11b and GFAP immunostainings to identify activated microglia and astrocytes, respectively (Figure 5A). There was a significantly increased number of CD11b+ cells in the CC of CPZ-exposed mice compared to the CTL mice (*p* < 0.001) (Figure 5B). Alternatively, venlafaxine produced a dramatic dose-dependent reduction in the number of CD11b+ cells in the CPZ-exposed mice (*p* < 0.001). The number of GFAP+ cells was also significantly higher in the CPZ-exposed mice than in the CTL mice, whereas venlafaxine counteracted to the CPZ effect (*p* < 0.001) (Figure 5C). No dose-dependent effect was observed.

## Discussion

Venlafaxine is a SNRI antidepressant that increases the equimolar concentrations of 5-HT and NE at neuronal terminals (Polluzi et al., 2013; Magalhães et al., 2014). Venlafaxine also has neuroprotective and anti-inflammatory effects in the CNS and peripheral nervous system (Tynan et al., 2012; Galecki et al., 2018). Moreover, a few clinical studies reported the cognitive effect of venlafaxine in patients with MDD (Tian et al., 2016; Cristancho et al., 2018). Nevertheless, it is difficult to determine whether cognitive improvements due to venlafaxine in MDD are due to the improvement of cognitive processes *per se* or because of improvement of depressive symptoms as well, because the severity of cognitive impairment is highly associated with the severity and subtypes of MDD (Snyder, 2013). Thus, the purpose of this research is to explore venlafaxine effect on cognitive and depression-like processes with respect to demyelination and inflammatory processes in the brain. The CPZ-induced demyelination mouse model was utilized to investigate cognitive impairments and mood symptoms associated with OL cell death and acute demyelination and associated neuroinflammation (Sun et al., 2017). Y-maze testing evaluated the cognitive effects of venlafaxine on CPZ-induced demyelination; a series of FST and TST behavioral tests assessed depression-like behaviors.

Notably, no depression-like behaviors were observed in all groups in the first FST following Y-maze test, suggesting that CPZ-induced cognitive impairment occurred in the absence of depression (Borsoi et al., 2015). Venlafaxine was found to improve working memory deficits in mice exposed to CPZ in a dose-specific manner. A high dose of venlafaxine attenuated the cognitive impairment of CPZ-treated mice; a lower dose of venlafaxine failed to reduce abnormalities in working memory. Venlafaxine’s effect on the cognitive process is likely independent to its effect on spontaneous depression. As it is accepted that carrying out FST or TST imposes significant stress to the testing animals, repeated expose to FST or TST is considered to generate a chronic depression model (Serchov et al., 2015). Mice receiving regular diet showed consistent immobility time across FST1-TST-FST2 processes, while CPZ-fed groups exhibited increased immobile time on TST and FST2. Venlafaxine treatment reversed the immobile time in the second FST. The results indicate that CPZ exposure for 5 weeks may not lead to an early depression-like behavior. However, demyelination and neuroinflammation caused by CPZ may alter the neurobiology of resilience and make animals more vulnerable to psychosocial stress (Osorio et al., 2017). Venlafaxine’s cognitive benefit may be partially due to its protective effects against chronic psychosocial stress (i.e., repeated exposure to stressors – FST and TST).

Venlafaxine also improved myelin integrity on CPZ-induced demyelination. Again, the effect was dose specific, a higher dose more beneficial than a lower one. In consistent with previous findings (Matsushima and Morell, 2001), CPZ feeding for 5 weeks induced OLs apoptosis and acute demyelination. On the contrary, high dose of venlafaxine reduced demyelination in the CC in the cortical areas. Venlafaxine’s effect on myelination mirrored the Y-maze results, suggesting that cognitive improvement can be associated with venlafaxine’s therapeutic effect on myelin integrity. Venlafaxine’s antidepressant effect seems independent to its myelin protection and anti-inflammatory effect.

Both mature, myelin-producing OLs and immature cell lines (OPC) belong to OL lineage and have a crucial role in myelination. OPCs proliferate and migrate to repair demyelinated lesion sites in MS and MDD (Kotter et al., 2006). Remyelination can be affected due to a hindered differentiation of these immature cells into myelin-producing, mature OLs (Lampron et al., 2015). Additionally, OPCs are capable of differentiating into mature OLs and astrocytes (Dimou and Gallo, 2015). These cells are the primary source of myelin repair and are upregulated following OL cell death (Matsushima and Morell, 2001) (VonDran et al., 2011).

Venlafaxine protected OL lineage cells in the present study. Specifically, a high dose of venlafaxine attenuated the depletion of mature OLs (i.e., Olig2+/GST-π+ cells) observed in the CPZ group. A high dose of venlafaxine also normalized compensatory increase of immature (i.e., NG2+) cells. Thus, the attenuated NG2+ cell numbers in CPZ + VEN20 groups may be due to preventing mature OL death as opposed to promoting the differentiation of OPCs.

A protective role of venlafaxine may also be attributed to reducing neuroinflammatory processes. Microglia, resident immune cells of the CNS, may polarize into a pro-inflammatory M1 phenotype or regenerative M2 phenotype (Karamita et al., 2017). Increased microglial expression in the CPZ model acts as a marker for neuroinflammation (Zhang et al., 2008). It is no surprise that a high dose of venlafaxine significantly reduced the number of CD11b (i.e., microglia) and GFAP (i.e. astrocytes) signal in the present study. Venlafaxine was reported to possess strong immunoregulatory activities (Galecki et al., 2018). Its ability to modulate microglia and macrophage activation in animal models of neurological disorders was also reported (Zychowska et al., 2015). Venlafaxine’s anti-inflammatory effect found that this study aligns with earlier reports (Mansouri et al., 2018). Further work is required to elucidate this mechanistic relationship; however, in the present study, we provide original evidence regarding the role of venlafaxine on attenuating microglia-mediated inflammation and demyelination.

## Summary

Here, we provide the evidence, for the first time, about a comparable protective effect of venlafaxine on myelin integrity and cognitive function in a demyelination mouse model. This study suggests that venlafaxine can be a medication of choice for depression and anxiety in MS. Venlafaxine’s strong neuroprotective and anti-inflammatory effects on myelin and OLs may provide extra therapeutic benefits to MS patients by altering the inflammation and demyelination process. Clinical studies with MS patients are needed to validate the findings and assess psychological and pathological progress.

## Ethics Statement

All animal procedures were performed in accordance with Canadian Council on Animal Care (CCAC) guidelines and were approved by the University Committee on Animal Care and Supply (UCACS), University of Saskatchewan.

## Author Contributions

All authors have participated and made substantial contributions to this paper. YZ and XB contributed in designing the study, conducting the experiments, and collecting the data. OA contributed to the data analysis and interpretation and writing of the manuscript. JW, AM, JC, and ZW performed part of the data analysis. FW, X-ML, and YZ contributed to the study design and manuscript revisions. All authors contributed to and had approved the final manuscript.

### Conflict of Interest Statement

The authors declare that the research was conducted in the absence of any commercial or financial relationships that could be construed as a potential conflict of interest.
